# Virus-Specific Nanobody-Chimeras Degrade the Human Cytomegalovirus US28 Protein in CD34+ Cells

**DOI:** 10.3390/pathogens13100821

**Published:** 2024-09-24

**Authors:** Emma Poole, Janika Schmitt, Stephen C. Graham, Bernard T. Kelly, John Sinclair

**Affiliations:** 1 Department of Medicine, University of Cambridge, Addenbrooke’s Hospital, Hills Road, Cambridge CB2 0QQ, UK; 2 Department of Pathology, Division of Virology, University of Cambridge, Addenbrooke’s Hospital, Hills Road, Cambridge CB2 2QQ, UK; 3 Cambridge Institute for Medical Research, Keith Peters Building, Hills Road, Cambridge CB2 0XY, UK

**Keywords:** human cytomegalovirus, latency, nanobodies, targeted degradation, proteostasis

## Abstract

After primary infection, human cytomegalovirus (HCMV) establishes lifelong persistence, underpinned by latent carriage of the virus with spontaneous reactivation events. In the immune-competent, primary infection or reactivation from latency rarely causes disease. However, HCMV can cause significant disease in immune-compromised individuals such as immune-suppressed transplant patients. Latency, where the viral genome is carried in the absence of the production of infectious virions, can be established in undifferentiated cells of the myeloid lineage. A number of stimuli can cause virus reactivation from latency to occur, beginning with the induction of viral immediate-early (IE) lytic gene expression. The suppression of viral IE gene expression to establish and maintain latent infection is known to result from a balance of viral and cellular factors. One key viral factor involved in this is the G protein-coupled receptor US28. Recently, we have shown that US28 is targeted for degradation by a modified nanobody (PCTD-Vun100bv) based on the novel PACTAC (PCSK9-antibody clearance-targeting chimeras) approach for targeted protein degradation. Furthermore, we have shown that this PCTD-Vun100bv-induced degradation of US28 results in IE gene expression in experimentally latently infected CD14+ monocytes. However, HCMV also establishes latency in CD34+ bone marrow cells, the progenitors of CD14+ cells. Here, we show that PCTD-Vun100bv also causes US28 degradation in these CD34+ primary cells, again resulting in the induction of viral IE gene expression. Additionally, we show that PCTD-Vun100bv can target US28 in naturally latently infected CD14+ monocytes from an HCMV-seropositive donor, allowing these latently infected cells to be killed by HCMV-specific cytotoxic T cells from that same donor. These observations support the view that targeting US28 for degradation during natural latency could be a tractable ‘shock-and-kill’ strategy to target the latent HCMV reservoir in myeloid cells.

## 1. Introduction

Human cytomegalovirus (HCMV) is a herpesvirus that infects 60% to 90% of the global population [1]. This universality results, at least in part, from a range of viral immune evasion functions that prevent the virus from being cleared after primary infection. The virus then establishes a life-long latent infection that helps the virus to avoid immune surveillance but is capable of sporadic reactivation [2]. HCMV causes little disease in the immune-competent but can cause significant disease in the immune-suppressed, resulting in a substantial clinical and economic burden [3]. Similarly, primary infection or reactivation during pregnancy can have serious effects on the developing foetus [4]. HCMV disease is often caused by reactivation from latency in the absence of a competent immune system that would otherwise control the virus [5]. Consequently, one suggested strategy to control HCMV reactivation is to target the latent reservoir by stimulating untimely virus reactivation, which, in the presence of a competent immune system, will be targeted and killed—the so-called ‘shock-and-kill’ approach [6,7].

After primary infection, HCMV can establish latency in bone-marrow-resident CD34+ progenitor cells [8,9]. As these cells exit the bone marrow and terminally differentiate into cell types, such as dendritic cells, HCMV reactivates, leading to the full temporal cascade of viral lytic gene expression, comprising immediate-early (IE), early (E) and late (L) genes [10]. During latency, the major immediate-early promoter (MIEP) is associated with repressive epigenetic histone marks, suppressing IE gene expression. In contrast, the induction of IE gene expression leading to virus reactivation is associated with epigenetic activation such as histone acetylation of the MIEP. The ability to control the MIEP by epigenetic modifiers has lent itself to efficient shock-and-kill strategies that target the latent reservoir [11,12,13,14,15,16,17,18].

Viral lytic gene expression is generally repressed during latency, but other viral genes are actively transcribed. While it is not fully clear how the expression of these so-called latency-associated transcripts is regulated, it may include the recruitment of the myeloid transcription factor GATA2 to the latency-associated gene promoters [12,19]. Interestingly, the latency-associated viral G protein-coupled receptor US28 is known to be required to maintain the latency-associated repression of the MIEP [20,21,22,23,24]. In the absence of US28, IE gene expression fails to be repressed. Consequently, another shock-and-kill strategy has involved driving IE gene expression by targeting US28. We have previously shown that the treatment of experimentally latently infected monocytes with a partial inverse agonistic nanobody of US28, Vun100bv, drives IE gene expression [25]. Furthermore, we have shown that a modified Vun100bv nanobody tagged with a degradation domain (PCTD-Vun100bv) degrades the US28 protein, again driving IE gene expression [26]. PCTD-Vun100bv is based on the novel PACTAC (PCSK9-antibody clearance-targeting chimeras) approach for targeted protein degradation. Furthermore, PCTD-Vun100bv treatment makes these otherwise latently infected CD14+ monocytes expressing IE protein targetable by HCMV-specific T cells (‘shock-and-kill’) [26]. However, HCMV also establishes latency in the CD34+ myeloid precursors of monocytes. While we have shown that PCTD-Vun100bv nanobody treatment of the CD34+ Kasumi-3 cell line causes US28 degradation and IE upregulation, it has not been analysed whether such targeted degradation of US28 also causes IE reactivation in bona fide primary CD34+ cells. Here, we show that the PCTD-Vun100bv treatment of experimentally latent Kasumi-3 cells causes US28 degradation and recapitulate that PCTD-Vun100bv induces IE gene expression in Kasumi-3 cells. Next, we show that PCTD-Vun100bv also induces IE gene expression in experimentally latent CD34+ primary cells. Additionally, PCTD-Vun100bv leads to the expression of IE genes in naturally latent primary CD14+ cells, resulting in their recognition and killing by HCMV-specific T cells. Taken together, these data show that PCTD-Vun100bv can be used to target the latent HCMV reservoir in primary CD34+ cells and CD14+ monocytes that are naturally latently infected with HCMV.

## 2. Materials and Methods

### 2.1. Primary Cells, Cell Lines, and Viruses

Primary CD34+ cells were commercially sourced (Lonza, Cambridge, UK), resuscitated, and maintained as per the manufacturer’s instructions. Kasumi-3 cells were maintained in RPMI (SLM-240-B, Merck, Gillingham, UK) supplemented with 10% sterile-filtered, heat-inactivated foetal bovine serum (TMS-016-B, Merck) and penicillin (100 U/mL) plus streptomycin (100 μg/mL) (516016, Merck). Human foreskin fibroblasts (HFFs) were maintained in DMEM supplemented with 10% sterile-filtered, heat-inactivated foetal bovine serum and penicillin (100 U/mL) plus streptomycin (100 μg/mL) (Merck). CD14+ primary cells were isolated from venous blood, as previously published [27], and were maintained in X-Vivo 15 (02-060Q, Lonza) with 2.5 mM additional L-glutamine (25030081, Gibco, Thermo Fisher Scientific, Crawley, UK). All human samples were obtained under ethical approval by, and after approval of, protocols from the Cambridgeshire 2 Research Ethics Committee (REC reference 97/092) and conducted in accordance with the Declaration of Helsinki. Informed written consent was obtained from all the volunteers included in this study before providing blood samples, and all experiments were carried out in accordance with the approved guidelines. The HCMV viruses, TB40E-IE2-eYFP and TB40E-US28, have been previously published [17,26].

### 2.2. PMA and Cytokine Treatments of Cells and IE Gene Expression Analyses

To detect the activation of IE gene expression in experimental latency settings, the TB40E-IE2-eYFP virus was used, which expresses YFP conjugated to the IE2 protein. Latently infected cells (primary CD34+, primary CD14+, or Kasumi-3 cells) were differentiated with cytokines or Phorbol-12-myristate 13-acetate (PMA) (P1585, Sigma, St. Louis, MO, USA) either as described in the text or as previously described [27]. The induction of IE gene expression could then be assessed by directly counting IE2-eYFP-positive cells. In the case of natural latency, IE was detected after induction from latency using immune fluorescence, as previously described [27].

### 2.3. T Cell Killing Assays

The T cell killing assays were carried out as previously published [6]. In short, CD14+ monocytes were isolated via CD14+ magnetic beads (130-050-201, Milltenyi, Bergisch Gladbach, Germany) from a seropositive donor prior to treatment with nanobodies, as described in the figure legends and text; the T cells were isolated by negative selection (130-096-535, Milltenyi). Post-treatment with nanobodies, IE-positive, treated monocytes were killed by co-culture with autologous T cells. After the removal of the T cells, cells were differentiated with cytokines (IL-4, 130-095-373; GM-CSF, 130-095-862, and LPS (L2630, Sigma)) and then co-cultured with HFFs. Positive foci of infection were enumerated, as described previously [27]. In short, co-cultured cells were fixed with 1% PFA/PBS for 20 min and then in 100% ethanol for 1 h at −20 °C. After this time, cells were washed in PBS and stained with anti-IE antibody (mouse monoclonal, mab810, Chemicon, Tokyo, Japan) at 1 in 2000 for 1 h RT and then washed with PBS prior to secondary staining (goat anti-mouse-FITC, ab6785, Abcam, Cambridge, UK) at 1 in 2000 at RT for 45 min with Hoechst (Catalogue number 14533, Sigma) nuclear staining.

### 2.4. Nanobodies

Nanobodies used in this study were generated as described previously [26].

### 2.5. Western Blot and Densitometry

Cell lysates were harvested in a Laemmli buffer (BioRad, Hercules, CA, USA) and analysed for Western blotting, as previously published [22]. They were then probed for the presence of actin (anti-actin goat primary AbCam, ab8229 1 in 2000) or GFP-US28 (anti-GFP rabbit primary AbCam, ab6556 1 in 1000) followed by HRP anti-goat or anti-rabbit secondary antibody (AbCam 1 in 2500). Where densitometry was calculated, Image J freeware was used to obtain and subtract the negative background value from all other values. Data were plotted relative to actin control.

## 3. Results

### 3.1. CD34+ Primary Cells and Kasumi-3 Cells Can Be Differentiated with PMA to Induce HCMV Reactivation

**Figure 1:**
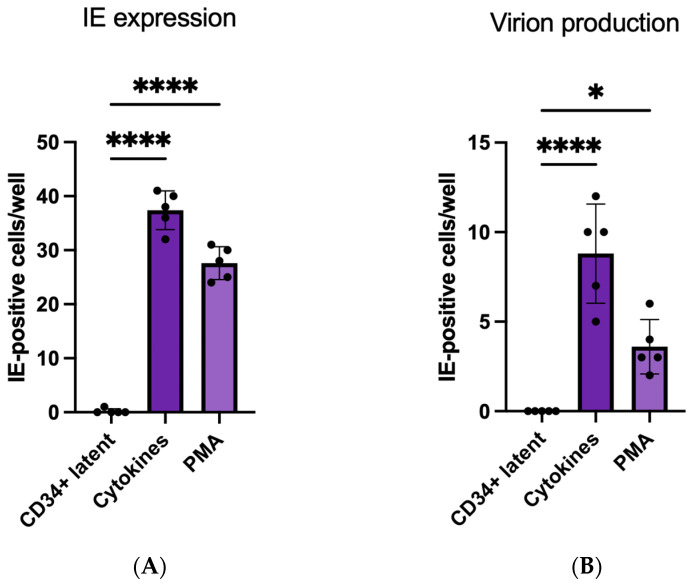
PMA or cytokine treatment of latently infected primary CD34+ stem cells results in full virus reactivation. Primary CD34+ cells were latently infected with TB40E-IE2-eYFP for 4 days before inducing differentiation with either TGF-beta (0.5 ng/mL), Flt-3Ligand (100 ng/mL), GM-CSF (100 ng/mL), TNF-alpha (2.5 ng/mL) and Stem Cell Factor (20 ng/mL) for 6 days before treating with LPS (50 ng/mL) for 3 days when the IE-positive cells were enumerated (**A**); next, the supernatant was transferred to fibroblasts for 3 days before again enumerating the IE-positive cells (**B**); alternatively, following the establishment of latency for 4 days, CD34+ cells were treated with PMA (20 ng/mL) for 7 days. IE-positive cells were enumerated (**A**); then, the supernatants were transferred to fibroblasts for a further 3 days before again enumerating IE-positive cells (**B**). All data are shown as individual data points, mean, and SD. Representative figures from two biological (different experiment) replicates are shown. A one-way ANOVA with Dunnett’s multiple comparisons test were used to determine statistical significance, where *p* < 0.05 = *. *p* < 0.0001 = ****.

The study of HCMV latency and reactivation can be modelled in a number of CD14+ and CD34+ cell lines and primary cell types, allowing for the analysis of the molecular mechanisms regulating the differentiation-dependent latent–lytic switch [7,12,16,17,28,29,30,31,32]. In this study, we used both a CD34+ cell line (Kasumi-3 cells) and a primary CD34+ cell model of virus latency. We used Phorbol-12-myristate 13-acetate (PMA) [13] to differentiate these cells, which acts as a surrogate for a mixed cocktail of cytokines (Figure 1).

To study and compare the effects of US28 targeting on latency in different CD34+ cell models of HCMV latency, we initially tested whether PMA, which is known to reactivate HCMV from latently infected Kasumi-3 cell lines, could also be used to reactivate primary CD34+ stem cells from latency [13,33]. Figure 1 shows that, following the establishment of latency, PMA induces reactivation of full productive infection from latently infected CD34+ primary cells.

### 3.2. HCMV-Infected CD34+ Primary Cells and Cell Lines Treated with PCTD-Vun100b Nanobody Fail to Establish Latency, Resulting in IE Expression but Not Full Virus Reactivation

**Figure 2:**
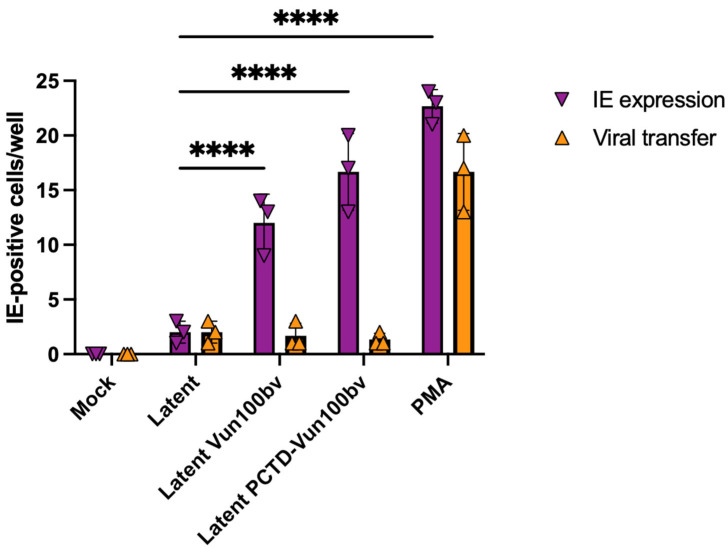
HCMV-infected Kasumi-3 cells treated with Vun100bv and the modified PCTD-Vun100b nanobody fail to establish latency, resulting in IE expression but no full viral reactivation. Kasumi-3 cells were isolated, left uninfected (mock), infected with HCMV-IE2-eYFP, and were left untreated (latent) or were treated with Vun100bv (200 nM), PCTD-Vun100bv (200 nM) or PMA (20 ng/mL) at 2 h post-infection. IE-positive nuclei were counted three days post-treatment (purple bars). Kasumi-3 cells were isolated, infected with HCMV-IE2-eYFP, and were left untreated or were treated with Vun100bv (200 nM), PCTD-Vun100bv (200 nM), or PMA (20 ng/mL) at 2 h post-infection. Four days post-treatment, the supernatant was transferred onto fibroblasts. IE-positive cells were counted 3 days after supernatant transfer (orange bars). Statistical analyses were performed using a two-way ANOVA with Tukey’s multiple comparisons test. All data are shown as individual data points, mean, and SD. Representative figures from two biological replicates are shown. *p* < 0.0001 = ****.

We have previously shown that infected CD14+ monocytes or the CD34+ Kasumi-3 cell line treated with the US28-degrading PCTD-Vun100bv nanobody show high levels of IE gene expression and fail to establish latency; in monocytes, this was correlated to US28 degradation [26]. However, this effect of targeting US28 with PCTD-Vun100bv has not been analysed in primary CD34+ hematopoietic progenitor cells. Consequently, we tested whether US28 expression in latently infected primary CD34+ cells could be targeted for degradation by PCTD-Vun100bv and whether this led to an inability to establish latency. Initially, we recapitulated the findings that the nanobody Vun100bv (a partial inverse agonist of US28) and PCTD-Vun100bv cause induction of IE gene expression but not full virus reactivation in Kasumi-3 cells. Figure 2 shows that, when using the same concentration of Vun100bv or PCTD-Vun100bv, IE gene expression was stimulated by both nanobodies (purple bars); however, this did not result in full virus reactivation, as assessed by infectious virion production (orange bars).

**Figure 3:**
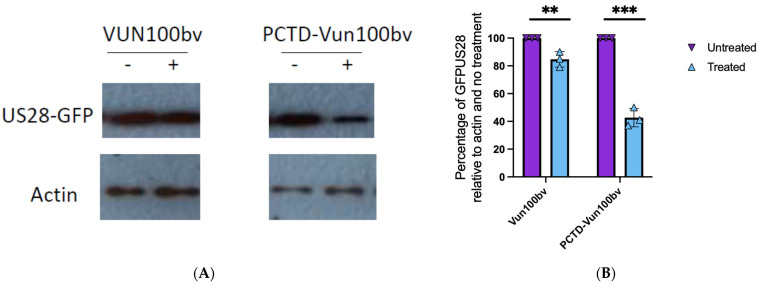
PCTD-Vun100bv causes degradation of US28 in a CD34+ cell line. Kasumi-3 cells were infected with TB40E-GFPUS28 virus for 3 days before treating with Vun100bv (500 nM) or PCTD-Vun100bv (500 nM) for 2 h and then harvested for Western blotting with anti-actin and anti-GFP (**A**); bands from Western blots were then analysed by densitometry and plotted relative to the actin control and untreated cells, where the graph represents individual and mean values from triplicate samples with standard deviation error bars (**B**). Statistical significance was determined using an unpaired *t*-test, where *p* < 0.01 = **. *p* < 0.001 = ***.

We also conducted a Western blot analysis for US28 levels in Kasumi-3 cells infected for 48 h with TB40E-US28GFP and treated for 24 h with PCTD-Vun100bv or Vun100bv. We observed a clear degradation of US28 following treatment with PCTD-Vun100bv. The degradation of US28 after Vun100bv treatment was, in contrast, less pronounced (Figure 3).

**Figure 4:**
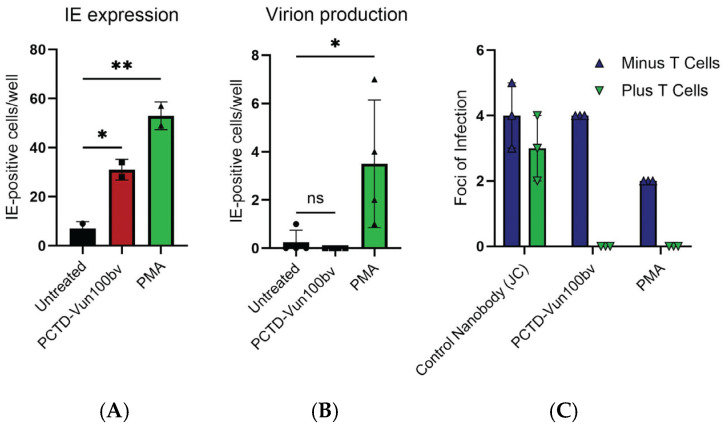
PCTD-Vun100bv induces IE gene expression in CD34+ cells and leads to the killing of naturally latent CD14+ monocytes. CD34+ cells were infected with HCMV-IE2-eYFP and were left untreated or were treated with PCTD-Vun100bv (500 nM) or PMA (20 ng/mL) 2 h post-infection. IE-positive cells were counted 2 days post-infection (**A**); at three days post-infection, the supernatant was transferred onto human fibroblasts. IE-positive nuclei were counted three days later (**B**). Statistical analyses were performed using one-way ANOVA followed by Dunnett’s multiple comparison test (**A**) or the Kruskal–Wallis test followed by Dunn’s multiple comparison test (**B**). All data are shown as individual data points, mean, and SD. Representative figures from two biological replicates are shown. *p* ≥ 0.05 = ns, *p* < 0.05 = *. *p* < 0.01 = **. Finally, CD14+ cells were isolated from a seropositive individual and treated with PMA or the indicated nanobodies (JC (control) or PCTD-Vun100bv). After 2 days, cells were co-cultured with autologous T cells (green) or left untreated (blue) for a further 2 days before differentiation and co-culturing with HFF cells. Cells were fixed and stained for IE, and foci of infection were counted. The graph represents one experiment of triplicate samples (**C**).

Having shown that PCTD-Vun100bv leads to the degradation of US28 and to the induction of HCMV IE gene expression in infected Kasumi-3 cells, we asked whether these effects were also observed in primary CD34+ cells. To do this, primary CD34+ cells were infected with HCMV TB40E-IE2-eYFP (which marks cells undergoing lytic infection) and then treated with Vun100bv or PCTD-Vun100bv or with the positive control, PMA. IE expression was assessed by counting the number of IE2-eYFP-positive cells. Figure 4A shows that treatment with either PCTD-Vun100bv or PMA leads to the upregulation of IE gene expression. However, whilst PMA treatment led to full virus reactivation, treatment with PCTD-Vun100bv, as was expected, did not (Figure 4B).

### 3.3. PCTD-Vun100bv Leads to IE Gene Expression in Naturally Latent Primary CD14+ Monocytes, Resulting in T Cell-Mediated Killing

Previously, we have shown that the PCTD-Vun100bv treatment of monocytes experimentally infected with PCTD-Vun100bv leads to transient IE gene expression and that the recognition of cells transiently expressing IE genes by HCMV-specific T cells leads to their death [25,26]. However, we wanted to test whether such immune targeting of naturally latently infected CD14+ cells with PCTD-Vun100bv was also possible. To do this, CD14+ monocytes were isolated from the total peripheral blood mononuclear cells (PBMCs) of a seropositive individual and then treated with PCTD-Vun100bv, a non-targeting US28 nanobody control nanobody (JC), or PMA. CD14+ cells were then co-cultured with autologous T cells from that same individual. After removing the T cells, the CD14+ cells were induced to reactivate with GM-CSF and IL-4, followed by LPS, as previously described [27]. The levels of surviving HCMV-infected CD14+ cells were assessed by the formation of foci of infection in fibroblast co-cultures. Figure 4C shows that naturally latently infected cells treated with PCTD-Vun100bv showed no infectious foci of infection, consistent with the view that naturally latent cells treated with PCTD-Vun100bv show a transient increase in IE gene expression. These IE-expressing cells can then be recognised and killed by the autologous T cells, resulting in few to no residual latent cells (Figure 4C).

Taken together, the data presented here show that US28 can be targeted for degradation using PCTD-Vun100bv in CD34+ cells latently infected with HCMV. Furthermore, PCTD-Vun100bv can induce IE gene expression in these experimentally latently infected primary CD34+ cells. Our data also show that the treatment of naturally latent CD14+ cells with PCTD-Vun100bv makes them recognisable by HCMV-specific T cells, allowing them to be targeted and killed.

## 4. Discussion

Pan-specific histone deacetylase (HDAC) inhibitors and other chromatin modifiers that stimulate the untimely reactivation of viruses, such as human immunodeficiency virus (HIV) and HCMV, have long been advocated as a means to target latent virus reservoirs [6,34]. One potential problem with this approach is the off-target effects resulting from the concomitant chromatin modification of the non-infected cell population. However, this can be circumvented by targeting a viral function to induce reactivation. The US28 gene of HCMV lends itself to this approach, as US28 is essential to establish and maintain viral latency by its repression of viral IE gene expression [5,21,22,24].

We have previously targeted US28 to induce the untimely reactivation of HCMV in cells of the myeloid lineage by using partial inverse agonistic US28-specific nanobodies. We have also shown that modified US28-specific nanobodies that carry a clearance-targeting chimeras domain, based on the PACTAC approach, allow for the nanobody-mediated degradation of US28 protein [26]. The data presented in this paper further demonstrate the suitability of US28-degrading nanobodies as a potential ‘shock-and-kill’ strategy for the detection and removal of HCMV latently infected myeloid progenitor cells.

Whilst both Vun100bv and PCTD-Vun100bv can be used to mediate the same effect (induction of IE in otherwise latently infected cells and their subsequent killing by HCMV-specific T cells), one advantage of the PCTD-Vun100bv nanobody is that it causes the degradation of US28 rather than just inhibiting US28 signalling. Targeting US28 with partial inverse agonistic nanobodies, like Vun100bv, could be subject to the recycling of US28 from the nanobody/US28 complex. In contrast, the degradation of US28 would not allow for any such recycling of US28, and this could impact the effectiveness of the drug, particularly in an in vivo setting. Further analysis would be required to test the relative effectiveness of the respective nanobodies/nanobody conjugates. Furthermore, nanobodies exhibit a low immunogenicity risk profile, and other protein degradation mechanisms, such as proteolysis-targeting antibodies (PROTABs), are well-tolerated in vivo [35,36].

However, further in vivo toxicity data are needed to evaluate if PCTD conjugates would elicit an immune response in vivo.

The data presented here show that PCTD-Vun100bv can mediate the degradation of US28 during the experimental latency of the CD34+ Kasumi-3 cell line, and, by doing so, induce IE gene expression in cells that would normally be silenced for IE gene expression. This adds to the observations that this nanobody conjugate can target US28 for degradation in the experimental infection of CD14+ monocytes and fibroblasts. Therefore, US28 is a suitable target for lytic infection, as well as for latency in monocytes and their CD34+ precursors. The data also show that targeting US28 for degradation with PCTD-Vun100bv is effective at inducing IE in experimentally latently infected primary CD34+ cells. Importantly, PCTD-Vun100bv also targets US28 in naturally latent myeloid progenitor CD14+ cells, resulting in their recognition and killing by HCMV-specific T cells from that same donor, suggesting that this nanobody conjugate may be suitable as a potential therapeutic in clinical settings.

## Data Availability

All data are included in the manuscript.
